# Methicillin-Resistant *Staphylococcus aureus* Contamination of Frequently Touched Objects in Intensive Care Units: Potential Threat of Nosocomial Infections

**DOI:** 10.1155/2022/1023241

**Published:** 2022-05-21

**Authors:** Dharm Raj Bhatta, Sumnima Koirala, Abha Baral, Niroj Man Amatya, Sulochana Parajuli, Rajani Shrestha, Deependra Hamal, Niranjan Nayak, Shishir Gokhale

**Affiliations:** ^1^Department of Microbiology, Manipal College of Medical Sciences, Pokhara, Nepal; ^2^Department of Medical Microbiology, Nobel College, Kathmandu, Nepal

## Abstract

**Background:**

Bacterial contamination in intensive care units is an important risk factor associated with increasing incidences of nosocomial infections. This study was conducted to study the bacterial colonization on commonly touched objects of intensive care units and antibiotic resistance pattern of bacterial isolates.

**Methods:**

This study was conducted in different intensive care units of Manipal Teaching Hospital, Pokhara, Nepal. A total of 235 swabs were collected from surfaces of bed rails, monitors, door handles, IV stands, telephone sets, nursing stations, medicine trolleys, sphygmomanometers, wash basin taps, dressing drums, stethoscopes, pulse oximeters, ventilators, defibrillators, and stretchers. Isolation, identification, and antibiotic susceptibility tests of the bacteria were performed following standard microbiological techniques.

**Results:**

Of 235 samples, bacterial growth was observed in 152 samples. A total of 90 samples of *Staphylococcus aureus* were isolated from 235 samples. Most of the sampling sites included in this study were found contaminated with *S*. *aureus*. The highest number of *S*. *aureus* was cultured from the surface of bed rails. Of the total *S*. *aureus* isolates, 54.4% (49/90) were methicillin-resistant *Staphylococcus aureus* (MRSA). Vancomycin resistance was detected among 8.1% MRSA isolates (4/49). *Acinetobacter* species were the commonest Gram-negative bacterial isolate.

**Conclusion:**

Bacterial contamination of the objects/instruments of the ICU was recorded to be high. The most common contaminating bacteria were *S*. *aureus* with a high percentage of MRSA and emergence of VRSA. Periodic microbiological surveillance, detection of contaminated sites, and effective decontamination methods would minimize the colonization by potential pathogens and their transmission.

## 1. Background

Intensive care units (ICUs) are the most essential working spaces in a hospital setting. Patients admitted in ICU are at a greater risk of nosocomial infections. The reported incidence of nosocomial infections in patients in ICU is around 2 to 5 times higher than in the general wards [1]. Microbial agents colonizing the environment of ICU and healthcare workers (HCWs) are important sources of nosocomial pathogens. Bacterial agents from the hands of HCWs and the ICU environment have been associated with outbreaks of hospital-acquired infections [2–4].

A wide range of bacteria, fungi, and viruses have been associated with nosocomial infections in ICU. Potential bacterial pathogens include *Staphylococcus aureus*, *Klebsiella* species, *Escherichia coli*, *Pseudomonas aeruginosa*, *Acinetobacter baumannii*, and *Enterococcus* species. Methicillin-resistant *Staphylococcus aureus* (MRSA) has been significantly associated with nosocomial infections in developing countries [5–7]. Gram-positive bacteria including MRSA survive for many weeks on dry inanimate objects/surfaces of the hospitals [8]. The increasing prevalence of MRSA among the patients in ICU has been a matter of great concern even in countries where standard infection control measures are regularly implemented. The environmental contamination rate by MRSA in ICU varies from hospital to hospital depending on a number of factors such as ward setting, crowding, patients with MRSA infections, carrier rate among staff, hand hygiene, and other infection control practices. The reported MRSA infections among ICU patients are associated with extended stay, poor outcome, and higher mortality and morbidity [9]. Approximately, 20% of infected patients in ICU die from invasive MRSA infections [10].

Frequently touched surfaces, instruments, and objects such as bed railings, floor, stethoscopes, and door handles are reported to be more frequently and heavily contaminated with bacterial agents [11, 12]. The microbes involved tend to be more difficult to eradicate due to high prevalence of antibiotic resistance. This study was planned to assess bacterial contamination of inanimate objects which are frequently touched in critical care units. Despite increasing incidences of hospital-acquired infections in intensive care units, contamination monitoring in the hospitals of Nepal is poor. Bacteriological examination of frequently touched sites and identification of areas colonized by potential pathogens would help in formulating cleaning/disinfection strategies in critical care units to minimize nosocomial infections.

## 2. Materials and Methods

This hospital-based study was conducted in intensive care units of Manipal Teaching Hospital, Pokhara, Nepal, over a duration of four months (May 2021 to August 2021). Ethical approval was obtained from the Institutional Review Committee (IRC) of Manipal Teaching Hospital, Pokhara, Nepal (MEMG/446/IRC). Manipal Teaching Hospital has over 750-bed capacity and is a referral center of the western region of Nepal. The hospital has different intensive care units such as medical ICU, postoperative ICU, critical care unit, pediatric ICU, and neonatal ICU. Total beds available in medical ICU, surgical ICU, and critical care unit are 14, 10, and 8, respectively.

Information about routine cleaning/disinfection of various objects/instruments of ICU was obtained from different units. Healthcare professionals of ICU maintain standard hand hygiene protocols. The floor cleaning/disinfection procedures are performed by mopping two times a day using a detergent solution. Noninvasive instruments, nursing station, table tops, and other objects are periodically cleaned with 75% alcohol swabs. Invasive instruments are sterilized by autoclave or disinfected by Cidex as per the manufacturer's instruction.

### 2.1. Specimen Collection

A total of 235 swabs were obtained from surfaces of bed rails (*n* = 75), monitors (*n* = 32), door handles (*n* = 21), IV stands (*n* = 14), telephone sets (*n* = 11), nursing stations (*n* = 11), medicine trolleys (*n* = 11), sphygmomanometers (*n* = 10), wash basin taps (*n* = 10), dressing drums (*n* = 09), stethoscopes (08), pulse oximeters (*n* = 06), ventilators (*n* = 06), defibrillators (*n* = 06), and stretchers (*n* = 05). A majority of these sites are frequently touched either by healthcare professionals or patients. Samples were collected by rubbing sterile swabs moistened with peptone water.

### 2.2. Isolation and Identification of Bacteria

All the samples were inoculated in a nutrient broth and incubated at 37°C for 18–24 hours. A subculture from the broth was performed on 5% sheep blood agar and MacConkey agar. Culture plates were incubated at 37°C for 24–48 hours. The bacterial isolates were identified by standard bacteriological procedures such as colony morphology, Gram staining, biochemical reactions, and other phenotypic characteristics [13].

### 2.3. Antibiotic Susceptibility Test

An antibiotic sensitivity test was performed by the Kirby–Bauer disc diffusion method using Mueller–Hinton agar plates (HI media, Mumbai, India) [14]. The antibiotics tested are ciprofloxacin (5 *μ*g), penicillin (10 IU), gentamicin (10 *μ*g), erythromycin (15 *μ*g), clindamycin (2 *μ*g) trimethoprim sulfamethoxazole (1.25/23.75 *μ*g), ceftazidime (30 *μ*g), amikacin (30 *μ*g), and imipenem (10 *μ*g). Bacterial isolates which are resistant to minimum one agent in three or more than three antibiotic groups were categorized as multidrug-resistant (MDR) [15]. Methicillin-resistant *S*. *aureus* (MRSA) isolates were detected by the cefoxitin (30 *μ*g) disc diffusion method [14]. Minimal inhibitory concentration (MIC) of vancomycin was performed by the Epsilometer test (HI media, Mumbai, India) following CLSI guidelines [14].

### 2.4. Biofilm Detection

Detection of biofilm among *S*. *aureus* and MRSA isolates was tested by the standard microtiter plate method [16].

## 3. Results

A total of 235 swabs were collected from different sites. Bacterial growth was observed in 152 swabs on both blood agar and MacConkey agar plates, while 83 samples did not show bacterial growth. A total of 90 *S*. *aureus* isolates were cultured from 235 samples. Most of the sampling sites included in this study were found to be contaminated with *S*. *aureus* isolates. *Acinetobacter* species were the commonest Gram-negative bacteria. Other bacterial isolates were *Pseudomonas* species, members of family *Enterobacteriaceae*, *Staphylococcus* species (coagulase negative), *Enterococci*, *Micrococci*, non-diphtheriae *Corynebacterium*, and *Bacillus* species. Frequency of bacterial agents isolated from objects/instruments of the ICU is depicted in Table 1.

All the sampling sites included in this study were contaminated with *S*. *aureus* (except medicine trolleys), with the highest number from the surface of bed rails. Among the total *S*. *aureus*, 54.4% (49/90) were MRSA and 45.5% (41/90) were identified as MSSA. Details of sampling sites and *S*. *aureus* isolates are shown in Table 2.

**Figure 1:**
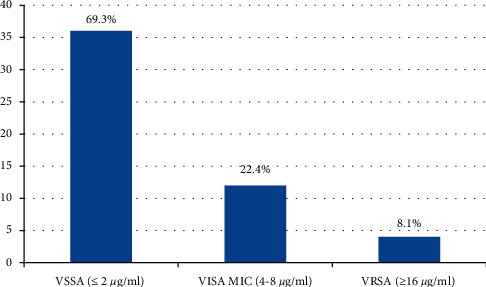
MIC results of vancomycin against MRSA isolates.

High percentages of *S*. *aureus* isolates were found susceptible to vancomycin, gentamicin, and ciprofloxacin. All the isolates of *S*. *aureus* were resistant to penicillin. Vancomycin resistance was detected among 8.1% MRSA isolates (4/49) with a minimal inhibitory concentration (MIC) value of >256 *μ*g/ml. Eleven isolates (11/49) of MRSA were intermediate-susceptible to vancomycin as shown in Figure 1. The antibiotic resistance patterns of *S*. *aureus*, MSSA, and MRSA isolates are shown in Table 3. The antibiotic resistance pattern of MRSA isolates to ciprofloxacin, cotrimoxazole, erythromycin, and gentamicin was significantly higher than that of MSSA as shown in Table 3. Among 90 *S*. *aureus* isolates, 20 (22.2%) were biofilm producers. No significant association was observed in biofilm-forming property of MSSA and MRSA isolates. *Acinetobacter* species were the commonest Gram-negative bacterial isolate (21/152). The drug resistance pattern of *Acinetobacter* species showed a high percentage of MDR isolates, with 38% (8/21) resistant to imipenem.

## 4. Discussion

Microbial colonization of objects/instruments in the ICU is considered a major factor for increased incidences of nosocomial infections. The reported prevalence of nosocomial infections in ICU in developing countries is 2–20 times higher than those of developed countries [17]. Nonadherence to standard hand hygiene protocols by healthcare professionals contributes significantly to the contamination of inanimate objects and cross-transmission during contact with the patient. *S*. *aureus* is one of the most common human pathogens and is significantly associated with nosocomial infections particularly in ICU. Increasing drug resistance among MRSA isolates and the emergence of vancomycin-resistant *Staphylococcus aureus* (VRSA) isolates have further exacerbated the problem. Identification of sites colonized by MRSA and other potential nosocomial pathogens would minimize the transmission among patients and thus help in reducing incidence of nosocomial infections in ICU.

In our study, bacterial contamination of frequently touched objects/instruments in ICU was high. The overall bacterial contamination rate in ICU was 64.7% (152/235) which is higher than that in findings of other studies [18, 19]. Some of the studies have reported higher percentage of bacterial contamination than our findings [20, 21]. This reflects that bacterial contamination rates vary from hospital to hospital within and outside the country. High rates of bacterial contamination in ICU could be associated with the admission of patients with different clinical conditions referred from various units, higher bed occupancy, prolonged stay, and poor compliances to infection control. Rates of bacterial contamination vary with frequency of use of life-supporting equipment, frequency of sterilization/disinfection, type and concentration of the disinfectant used, fumigation, and other infection control practices in ICU.

The findings of our study showed contamination of objects/instruments in ICU with a diverse group of Gram-positive and Gram-negative bacteria. In our study findings, contamination by Gram-positive bacteria was higher than that by Gram-negative bacteria. Similar findings are reported by other studies [19, 20]. In contrast to our findings, a study from India reported higher contamination rates with Gram-negative bacterial isolates [22].

*S*. *aureus* and MRSA are notorious nosocomial pathogens associated with a variety of clinical conditions in ICU. Colonized hands of healthcare workers account for 20–40% of infections due to cross contamination [23, 24]. All the sites included in the study were contaminated with *S*. *aureus* except the medicine trolley. Among Gram-positive bacteria, *S*. *aureus* was the most common potential pathogen isolated with 54.4% MRSA. Surfaces of bed rails yielded the highest number of *S*. *aureus* isolates as compared to other sites. Bed rails are the one of the most frequently touched surfaces by healthcare providers, patients, and visitors. Objects/instruments of different units of a hospital often remain contaminated with *S*. *aureus* due to its prolonged survival [8]. A study conducted in the environmental samples of neonatal ICU of Manipal Teaching Hospital reported *S*. *aureus* as one of the common potential pathogens with 33.3% MRSA isolates [25]. Patients admitted in ICU are often immunocompromised and vulnerable to nosocomial infection. Contamination of *S*. *aureus* and MRSA on these sites increases the risk of transmission among the patients and may result in septicemia and pneumonia.

Infections associated with MRSA are difficult to treat due to limited therapeutic options. Among MRSA isolates, 8.1% (4/49) were resistant to vancomycin. Detection of vancomycin-resistant isolates is a serious matter of concern and requires special attention. Various studies from Nepal have reported the emergence of VRSA isolates in clinical samples [26, 27]. Contamination of objects of ICU with VRSA poses a great risk of nosocomial infections. 22.4% (11/49) isolates of MRSA were intermediate-susceptible to vancomycin. This may be an alarming finding for increased incidences of VRSA infections in the near future.

Biofilm formation among *S*. *aureus* isolates was studied. Among *S*. *aureus* isolates, 22.2% (20/90) were biofilm producers. *S*. *aureus* isolates with biofilm-forming property can survive longer on hospital surfaces resulting in long-term survival and are a potential source of nosocomial infections. A recent study from Manipal Teaching Hospital reported a higher percentage (31.8%) of biofilm among *S*. *aureus* isolates cultured from inanimate objects of the hospital [24].

Among Gram-negative bacteria, potential pathogens isolated were *Acinetobacter* species, *Pseudomonas* species, and bacteria from family *Enterobacteriaceae*. In our study, *Acinetobacter* species were the most common Gram-negative bacteria isolated. *Acinetobacter* species are well-established pathogens among the patients admitted in ICU due to resistance to different groups of antibiotics and chemical disinfectants. A study from Manipal Teaching Hospital reported MDR *Acinetobacter* species as the most common bacterial pathogen associated with lower respiratory tract infections among ICU patients [28]. Contamination of objects/instruments of ICU with *Acinetobacter* species is an additional risk factor of nosocomial pneumonia. Drug resistance pattern showed that a high percentage (38%) of isolates was resistant to imipenem. Resistance to imipenem is alarming and challenging to clinicians for therapeutic management. Increasing resistance to higher-generation antibiotics limits the treatment options with additional financial burden and long-term hospitalization among the patients.

Our study findings are important to generate awareness among infection control team and healthcare professionals regarding contamination of ICU with bacterial agents and their possible role in nosocomial infections. This was a single center study, and findings may not be generalized. We did not study the association between contamination of objects/instruments and nosocomial infections. Molecular characterization of the isolates was not performed.

## 5. Conclusion

Our study results showed high level of bacterial contamination of the frequently touched objects/instruments of ICU. Isolation of MRSA and VRSA from the sites is a potential threat of nosocomial infections. The present study emphasizes need for modification in the existing cleaning/disinfection procedures in order to minimize the contamination by potential pathogens. Periodic microbiological surveillance of the ICU environment with effective infection control practices is expected to minimize the bacterial contamination and transmission. Gentamicin may be empirically used in suspected cases of staphylococcal infections in ICU.

## Figures and Tables

**Table 1:** Frequency of bacterial isolates colonized on objects of intensive care units.

Organism	Total isolates (*n* = 152)			Total
	ICU	CCU	SICU	
*Staphylococcus aureus*	31 (20.4%)	19 (12.5%)	40 (26.3%)	90 (59.2%)
Coagulase-negative *Staphylococci*	6 (3.9%)	3 (1.9%)	4 (2.6%)	13 (8.5%)
*Bacillus* species	2 (1.3%)	2 (1.3%)	3 (1.9%)	7 (4.6%)
*Micrococcus* species	3 (1.9%)	1 (0.6%)	2 (1.3%)	6 (3.9%)
Non-diphtheriae *Corynebacterium*	1 (0.6%)	3 (1.9%)	1 (0.6%)	5 (3.2%)
*Enterococcus* species	1 (0.6%)	—	—	1 (0.6%)
*Acinetobacter* species	6 (3.9%)	9 (5.9%)	6 (3.9%)	21 (13.8%)
*Pseudomonas* species	1 (0.6%)	1 (0.6%)	—	2 (1.3%)
*Escherichia coli*	—	2 (1.3%)	2 (1.3%)	4 (2.6%)
*Klebsiella pneumoniae*	—	1 (0.6%)	—	1 (0.6%)
*Enterobacter* species	—	—	1 (0.6%)	1 (0.6%)
*Proteus* species	1 (0.6%)	—	—	1 (0.6%)

ICU: intensive care units, CCU: critical care units, SICU: surgical intensive care units.

**Table 2:** Distribution of *Staphylococcus aureus* (MSSA and MRSA) isolated from environmental samples of intensive care units.

Sampling sites	Number of swabs	Number of *S*. *aureus* isolates	Number of MSSA isolates	Number of MRSA isolates
Bed rail	75	29	10	19
Monitor	32	8	3	5
Door handle	21	12	8	4
IV stand	14	8	5	3
Telephone set	11	5	4	1
Nursing station	11	5	2	3
Medicine trolley	11	0	0	0
Sphygmomanometer	10	5	2	3
Wash basin tap	10	3	1	2
Dressing drum	9	5	3	2
Stethoscope	8	2	0	2
Pulse oximeter	6	1	1	0
Ventilator	6	2	1	1
Defibrillator	6	3	0	3
Stretcher	5	2	1	1
Total	235	90	41	49

**Table 3:** Antibiotic resistance patterns of *S*. *aureus*, MSSA, and MRSA isolates.

Antibiotics	*S*. *aureus* isolates (*n* = 90) frequency (%)	MSSA isolates (*n* = 41) frequency (%)	MRSA isolates (*n* = 49) frequency (%)	*P* value
Ciprofloxacin	25 (27.7%)	6 (14.6%)	19 (38.7%)	0.017
Cotrimoxazole	33 (36.6%)	09 (21.9%)	24 (48.9%)	0.009
Clindamycin	65 (72.2%)	28 (68.3%)	37 (75.5%)	0.486
Cefoxitin	49 (54.4%)	00	49 (100%)	0.000
Erythromycin	77 (85.5%)	30 (73.1%)	47 (95.9%)	0.003
Gentamicin	15 (16.6%)	00 (0%)	15 (30.6%)	0.000
Penicillin	90 (100%)	41 (100%)	49 (100%)	—
Vancomycin	04 (4.4%)	00 (%)	04 (8.1%)	0.123

## Data Availability

Data generated in this study are included within this article.
